# Passive fit evaluation of implant superstructures by analyzing the accumulated screw tightening torque

**DOI:** 10.1186/s40729-026-00665-7

**Published:** 2026-01-14

**Authors:** Reiko Kobatake, Kazuya Doi, Hiroshi Oue, Tomoko Izumikawa, Kaien Wakamatsu, Kazuhiro Tsuga

**Affiliations:** Department of Advanced Prosthodontics, Hiroshima University Graduate School of Biomedical and Health Sciences, 1-2-3 Kasumi, Minami-Ku, Hiroshima City, Hiroshima 734-8553 Japan

**Keywords:** Dental implants, Prosthesis fitting, Prostheses and implants, Torque

## Abstract

**Purpose:**

Implants, particularly restorations with screw-retained superstructures, must have a stable passive fit. Clinical fit evaluations are often subjective. Therefore, this study investigated the feasibility of identifying misfits and evaluating the fit based on the screw-tightening torque.

**Methods:**

Screw-retained monolithic zirconia superstructures supported by two implants and several master models were prepared. A passive-fit model was created by connecting the implant body to the superstructure and embedding it in dental stone. Vertical misfit models were created by placing titanium membranes with thicknesses of 50, 100, 150, and 200 µm between the superstructure and implant body. In each model, the screw-tightening torque on each side was measured at 0.05-s intervals up to 25 N cm. The torque for the second screw was divided into initial, middle, and final rotation phases, and the results under different conditions were compared.

**Results:**

No vertical gaps were observed at the joints in the passive-fit model, whereas vertical gaps were observed in the misfit models. No significant differences between the accumulated torque on each side were observed in the passive fit group. By contrast, significant differences between the accumulated torque on each side were observed in the misfit groups. When the instantaneous torque was divided into three phases based on time, the samples with misfits of 150 and 200 µm showed high values in the middle and final rotation phases.

**Conclusions:**

Passive fit and misfit can be evaluated numerically and objectively by measuring the torque and accumulated torque during the installation of screw-retained superstructures.

## Background

The compatibility between implant superstructures and implant bodies or abutments significantly affects the long-term prognosis of implant treatments. Screw-retained superstructures are connected to multiple implant bodies, and the implant bodies and screws must be adapted and connected in a passive fit state. Passive fit indicates that this adaptation should be accomplished without tension on the retaining screws. Passive fit can be achieved by producing a well-fitting superstructure; however, misfits may occur owing to deformation of the impression or model or shrinkage during frame production. If the superstructure is screwed to the implant body or abutment in a misfit state, then the implant body will be subjected to continuous stress owing to distortion. In addition, gaps will form at the device interface, which will lead to microleakage and increase the risk of peri-implantitis. Several studies have reported that a misfit between the prosthesis and implant body can generate stresses in the bone surrounding the implant, as the misfit induces static loads on the implant body [1–3]. Unlike natural teeth, which can move approximately 100 μm within their sockets owing to the periodontal ligament, implants exhibit limited mobility of approximately 10 μm [4]. Therefore, prosthetic misfits in implant-supported restorations are likely to have more detrimental effects than those in tooth-supported prostheses. Technical and manufacturing limitations make it difficult to achieve a perfect passive fit, and misfits of 30–50 µm have been reported to be clinically acceptable [5]. However, several studies have reported that a misfit limit of 150 µm is sufficient to minimize the risk of biomechanical complications in clinical practice [6, 7]. Although reports regarding the acceptable size of the misfit vary, no reports allow misfits of 200 μm or more. Therefore, misfits should be considered to be at most 150 μm.

Several methods of evaluating the passive fit have been described. For example, the passive fit can be evaluated visually using methods such as one-screw tests or microscopy observations. Many studies have investigated the usefulness of one-screw tests, such as the Sheffield test; however, these tests have only been evaluated using master casts, and clinical evaluation is difficult owing to the presence of connections and surrounding tissues at the implant level. Other studies have shown that misfits can be confirmed via visual evaluation using a microscope. However, this method also relies on master casts and cannot be performed in clinical scenarios. In clinical practice, the screw-tightening sensation has been used to assess passive fit conditions; however, this method is affected by the experience of the operator, and objective evaluation cannot be achieved. Radiography is widely used for passive fit evaluation in clinical practice. However, factors such as the film holding and line angle are difficult to standardize, and subsequent differences can affect the evaluation. Furthermore, numerical evaluation of the misfit is difficult. Researchers have also attempted to evaluate the passive fit by using a surgical motor device to measure the torque. For example, Cardilini et al. evaluated the passive fit by measuring the torque during screw tightening [8]. Similarly, Figueras-Alvarez et al. recently reported that the passive fit can be evaluated by measuring the prosthetic screw tightening torque, which is suitable for objective evaluation [9]. These techniques can be applied clinically, and they allow objective numerical evaluation. Therefore, accurate torque measurements are expected to enable the evaluation of passive fit or misfit.

In a previous study, we demonstrated that the drilling torque can be measured accurately, even at low bone-density sites, using a torque-controlled surgical motor operated via a computer-based program [10, 11]. Furthermore, we reported a new method for evaluating the initial fixation using the peak insertion torque and accumulated values [12]. This method of recording the torque as an accumulated value is expected to be applicable to passive fit evaluation.

We hypothesized that the torque generated during screw tightening will be higher earlier in the case of a misfit than in the case of a passive fit, and the accumulated torque will increase in proportion to the magnitude of the misfit. Therefore, this study aimed to verify the usefulness of this approach as an objective method of evaluating the passive fit. Moreover, it shows that this method can be used to numerically evaluate the passive fit by creating a stage-specific misfit model and comparing the accumulated values of the superstructure tightening torque under passive fit conditions.

## Methods

### Materials

**Fig. 1:**
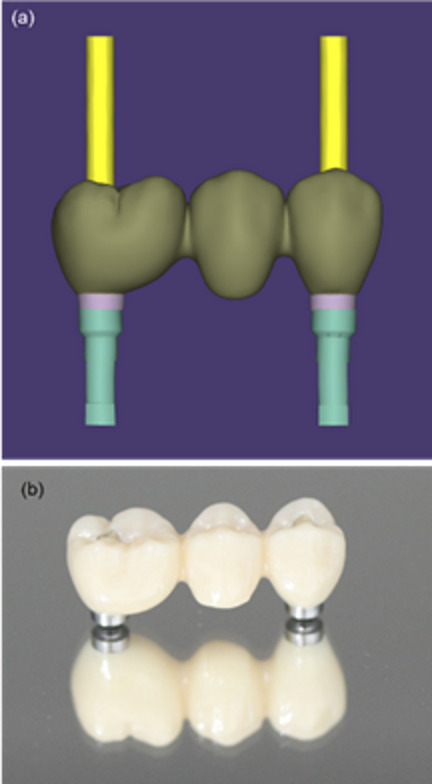
Superstructure. **a** Zirconia superstructure supported by two parallel implants 22 mm apart designed using computer-aided design. **b** Fabricated superstructure composed of a monolithic zirconia body and titanium base

A zirconia prosthesis supported by two implants was prepared using computer-aided design/computer-aided manufacturing (CAD/CAM) technology (Fig. 1), and a non-engaging titanium base was cemented on each side. Implant bodies with a diameter of 4.3 mm (NobelReplace®; Nobel Biocare, Kloten, Switzerland) were also prepared. A surgical implant motor system (Surgic Pro 2; Nakanishi Inc, Tochigi, Japan) was used for the test, and titanium thin films with thicknesses of 50, 100, 150, and 200 µm were used to prepare the misfit models.

### Simulation model fabrication

**Fig. 2:**
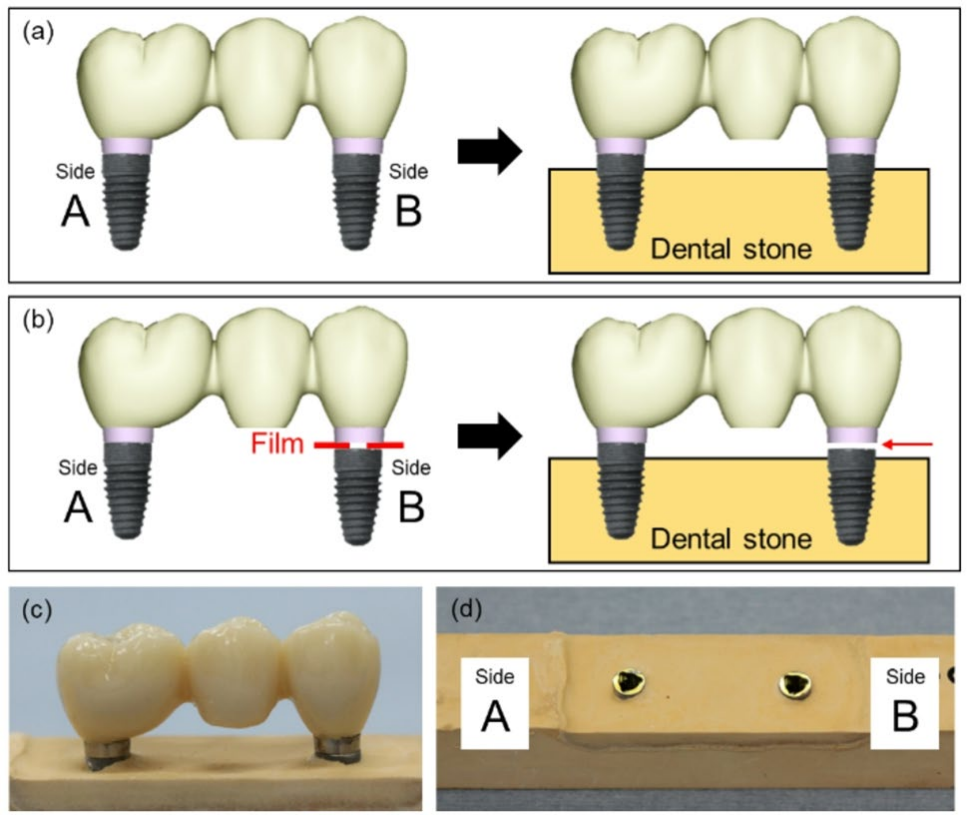
Passive fit and misfit models. **a** Passive fit model created by connecting the implant body (Side A and Side B) to the titanium base of the superstructure and then embedding it in dental stone. **b** Misfit models created by connecting the implant body to the titanium base, inserting titanium films of various thicknesses (50, 100, 150, and 200 µm) on side B, and then embedding them in dental stone. **c** Superstructure on a plaster model. **d** Implant body placed in the plaster model

A passive fit model was created as follows: First, implant bodies were connected to the molar and premolar sides (Sides A and B, respectively) of the prosthesis via abutment screws with a torque of 15 N cm. Next, the implant bodies were embedded and fixed in type IV dental stone (New Fuji Rock; GC Corp., Tokyo, Japan), which was prepared using the powder–liquid ratio specified by the manufacturer (Fig. 2a). Misfit simulation models were also created as follows: On Side A, the abutment screw was tightened to 15 N cm to create a fit condition. On side B, titanium thin films with thicknesses of 50, 100, 150, and 200 µm were inserted between the non-engaged titanium base of restoration and the implant body, and then the restoration screws were tightened to 5 N cm to reproduce vertical misfit conditions. Each misfit model was embedded in type IV dental stone (Fig. 2b). The four vertically simulated misfit conditions were labeled mis-50, mis-100, mis-150, and mis-200 according to the thickness of the titanium film. The connection conditions at Side B of each model were observed using a microscope (SZX7, OLYMPUS, Tokyo, Japan).

### Measurement

**Fig. 3:**
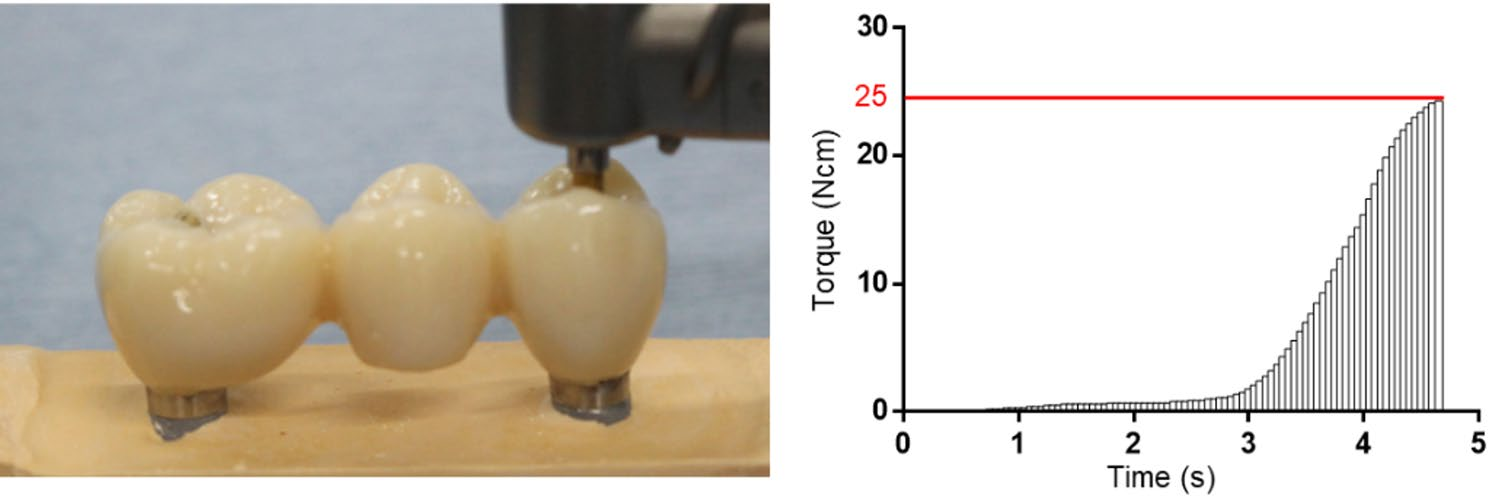
Measurement. **a** Measurement of the accumulated torque. **b** Accumulated torque waveform leading to the limit torque value

For each model, torque measurements were performed using the following procedure (*n* = 20): First, a screw was inserted on Side A without fastening. Using an implant motor with a rotation speed of 10 rpm and a limiter at 5 N cm, the screw was tightened until the specified torque was reached. Next, the screw was loosened by half a turn with a hand driver, and again using the implant motor with a rotation speed of 10 rpm and a limiter at 25 N cm, the screw was tightened until the specified torque was reached. During this process, the torque was recorded at 0.05-s intervals (detection sensitivity: less than 1 N cm). Next, the procedure was repeated for Side B, and the screw on Side A was kept tight. After tightening at 5 N cm, the screw was loosened by half a turn and then tightened again at 25 N cm. The torque was recorded under the same conditions used for Side A. Among the measured torques, the total torque generated until each screw reached the maximum torque was calculated as the accumulated torque for each side under each condition. Moreover, for Side B, the torque generated until the maximum torque was reached was divided temporally into three phases (initial, middle, and final rotation phases), and the results under the different conditions were compared. For both Side A and Side B, the time required to reach the peak torque value was recorded during each measurement. In addition, microscopic images of the interface between the implant and the titanium base on Side B were obtained with both screws tightened. Subsequently, only the screw on Side B was released, and microscopic imaging of the same interface was repeated under the same conditions (Fig. 3).

#### Statistical analysis

Comparisons of the accumulated torque values between Side A and Side B were performed using a paired t-test. Comparisons of torque values within Side B were performed using the one-way ANOVA with Tukey’s post-hoc test. A significance level of *p* < *0.05* was considered statistically significant.

## Results

**Fig. 4:**
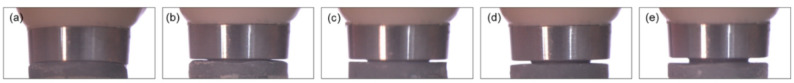
Microscopy images of the connections between the titanium bases and implant bodies

Microscopy images showed the connections between the implant bodies and titanium bases of the restorations on Side B (Fig. 4). No vertical gaps were observed at the connection in the passive-fit model (Fig. 4a). By contrast, vertical gaps were observed in the misfit models (Fig. 4b–d).

In the passive fit group, no significant differences between the accumulated torques at Sides A and B were observed. However, significant differences between the accumulated torques at Sides A and B were observed in the mis-50, mis-100, mis-150, and mis-200 groups (Table 1). The time required to reach the peak torque was 4.46 ± 0.52 s, 4.53 ± 0.52 s, 4.49 ± 0.50 s, 4.35 ± 0.43 s and 4.29 ± 0.40 s for the passive, mis-50, mis-100, mis-150 and mis-200 conditions, respectively, on Side A; corresponding values for Side B were 4.54 ± 0.31 s, 4.43 ± 0.15 s, 4.50 ± 0.22 s, 4.64 ± 0.16 s and 4.87 ± 0.41 s. There were no statistically significant differences among the conditions on Side A, indicating comparable times to reach the peak torque. In contrast, on Side B, the mis-200 condition showed significantly higher values than passive, mis-50 and mis-100 groups.

**Table 1:** Comparison of the accumulated torque at Side A and Side B

	Side A (Ncm)	Side B (Ncm)
Passive	391.0 ± 29.6	397.3 ± 23.9^a^
mis-50	376.0 ± 22.2	386.6 ± 20.6^b^
mis-100	371.6 ± 18.6	394.0 ± 20.0^c^
mis-150	391.3 ± 21.6	417.7 ± 29.0^d^
mis-200	398.3 ± 23.5	433.8 ± 20.1^e^

^a^*p* = 0.29, ^b^*p* = 0.03, ^c^*p* = 0.0007, ^d^*p* = 0.0002, ^e^*p* < 0.0001

**Fig. 5:**
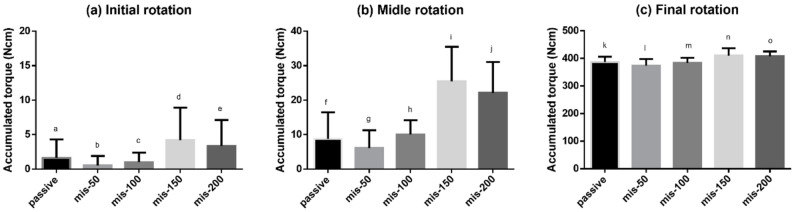
Accumulated torques at Side B over time in the different groups. **a** The initial rotation phase (1/3 point), no differences were observed between the passive fit and misfit groups. **b** The middle rotation phase (2/3 point), the mis-150 and mis-200 groups showed higher accumulated torques than other groups. Significant differences were detected between f and i (*p* < *0.0001*), f and j (*p* < *0.0001*), g and i (*p* < *0.0001*), g and j (*p* < *0.0001*), h and i (*p* < *0.0001*), and h and j (*p* < *0.0001*). **c** The final rotation phase (3/3 point), the mis-150 and mis-200 groups showed higher accumulated torques than the other groups. Significant differences were detected between k and n (*p* = *0.0068*), k and o (*p* = *0.0166*), l and n (*p* < *0.0001*), l and o (*p* < *0.0001*), m and n (*p* = *0.0011*), and m and o (*p* = *0.0032*)

The accumulated torque was compared over the time required to reach a tightening torque of 25 N cm (Fig. 5). Statistically significant differences were observed in all four groups analyzed using one-way ANOVA: *p* = *0.0008* (Fig. 5a), *p* < *0.0001* (Fig. 5b), *p* < *0.0001* (Fig. 5c). No significant differences were observed between the passive-fit and misfit groups during the initial rotation period. In the middle rotation phase, the mis-150 and mis-200 groups showed higher values than the passive-fit, mis-50, and mis-100 groups. In the final rotation phase, the mis-150 and mis-200 groups showed higher values than the passive-fit, mis-50, and mis-100 groups. A comparison of the accumulated torques showed similar results for the middle and final rotation phases.

**Fig. 6:**
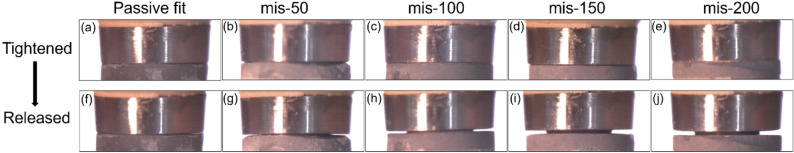
Stereomicroscope image of Side B screw: before and after releasing Side B screw. **a**, **f** No gap was observed under the passive-fit condition before/after loosening the Side B screw. **b**–**e** Pre-existing gaps at the implant–titanium base interface were disappeared by screw tightening in all misfit conditions. **g**–**j** Gaps reappeared after releasing the Side B screw

Under all misfit conditions, pre-existing gaps at the implant–titanium base interface were closed by side B screw tightening. After releasing the side B screw, these gaps became visible again in all misfit conditions. In contrast, no gap was observed under the passive-fit condition either before or after loosening (Fig. 6).

In the passive fit model, no gaps were observed at the joint. In the misfit models, gaps were observed at the joints, and the gaps became larger as the size of the misfit increased.

## Discussion

This study observed differences in the accumulated torques of the tightening screws in the misfit state models. Moreover, torque generation occurred earlier in the misfit groups than in the passive-fit group, and this tendency became more pronounced as the size of the misfit increased.

Previous reports mentioned the possibility of evaluating the passive fit based on the correlation between the torque and rotation speed; however, they did not measure the torque at detailed intervals to evaluate the fit [8, 9]. Rutkunas V et al. reported a method for evaluating the passive fit based on the screw rotation angle [13]. They determined that a misfit requires a larger screw rotation angle to reach the specified torque value using a digital torque wrench. These evaluation methods are clinically feasible and allow numerical evaluation. However, some problems remain. For example, the long intervals required for the torque measurements make it impossible to measure the torque in detail, and the magnitude of the strain cannot be evaluated based on the rotation angle. We believe that these problems can be addressed by performing detailed torque measurements. Detailed screw-tightening torque measurements, which record changes in the torque over time, have not been reported previously. In this study, a detailed torque measurement program with intervals of 0.05 s was pre-verified using bone density measurements.

Researchers generally accept that a perfect passive fit is difficult to achieve clinically. This is attributed to various factors, including impression-coping displacement, plaster expansion, and casting shrinkage during metal frame fabrication [14–16]. In this study, the torque on the side screws was measured by rotating them by half a turn from the point where resistance was detected when tightening by hand until a tightening torque of 20 N cm was generated. The measured instantaneous torque was divided into three phases based on the total measurement time, and the accumulated torque was calculated for the initial, middle, and final rotation phases. No significant differences were observed in the accumulated torques of the tightening screws on either side of the passive-fit model.

In this experiment, the implant body was connected to a restoration that was created in advance using CAD/CAM, and a passive-fit model was created during the process of embedding the implant into the plaster and creating the working tail. Therefore, these effects could be eliminated, and it can be said that an ideal passive fit model was achieved. In the passive-fit model, no significant differences were observed in the accumulated torque values for the tightening screws on Sides A and B. However, a slight difference was observed in the screw-tightening torques. This was thought to be due to the gaps in the joints of the non-engaged parts caused by lathe machining.

By contrast, in the misfit groups, significant differences were observed in the accumulated torques on the side screws. The accumulated torque on the screw on the non-conforming side was largest in the mis-200 group, and it increased in proportion to the size of the misfit. In the analysis of the torque accumulation over time, the passive-fit, mis-50, and mis-100 groups showed high torque accumulation in the latter half of the analysis period. In the mis-150 and mis-200 models, the torque increased in the middle phase. These results support the hypothesis that torque generation occurs earlier as the misfit increases.

After tightening the screws on Side B, the gap between the implant and the titanium base disappeared under all conditions. This is due to distortion of the superstructure by screw tightening, causing it to come into contact with the implant body. When comparing measurement times, the time was significantly longer under the mis-200 condition, and this is also thought to be due to distortion.

No fractures or other damage to the working cast were observed during the test period, suggesting that the distortion occurred somewhere in the zirconia crown, screw, or titanium base of the zirconia superstructure.

The increase in the accumulated torque during the middle phase of the tightening period is a meaningful result that supports the subjective evaluation of the resistance felt during hand tightening with a numerical evaluation. In this study, the evaluation of the mis-150 group was found to be equivalent to the evaluation method currently in use, suggesting its potential usefulness. Previous studies have shown that X-ray imaging can detect gaps of 70 μm [17]. However, in clinical practice, the accuracy of this method is easily affected by factors such as film retention and the angle of the irradiation tube [18]. Microscopy allows detailed observations of the fit; however, its use is limited to working models, and it is not suitable for clinical applications.

Detailed measurements of the accumulated torque, the main method in this study, can be evaluated in the same way as dental treatment, and this method does not involve exposure to radiation, and allows numerical comparison. In this study, non-engaging (NE)-type connections were used to connect the restoration to the implant body. In cases of single-tooth loss, engaging (E)-type connections with an anti-rotation mechanism are clinically recommended; however, in many cases, misfits may cause significant strain during screw fixation. Therefore, NE-type connections are recommended for cases with multiple devices. Rutkunas V et al. reported that, in cases of misfit, NE-NE connections result in less strain than NE-E or E-E connections [13]. This is because NE connections intentionally leave a gap between the abutment and the implant, allowing for a misfit.

In this study, no significant difference was observed between the accumulated torque on side B in the mis-50, mis-100, and passive-fit groups. This can be attributed to the NE-type joint mechanism, which resulted in less strain and no difference in the torque measurement evaluation. The lack of a significant difference between the mis-50 and mis-100 groups, in particular, may be explained by the approximately 20 μm design tolerance between the abutment and implant body, as described in previous reports, which could have mitigated the effects of the induced misfit [2, 17]. However, the fact that differences could be detected based on the accumulated torque when the misfit was 150 μm or more suggests that this method may also be applicable to NE-NE restorations, which are widely used in clinical practice. Furthermore, if the accumulated torque value measured in this study can be used to detect misfit, it may enable the objective assessment of internal stresses caused by misfit. This could, in turn, serve as a clinically applicable tool to prevent future mechanical and biological complications.

The limitations of this study are as follows: There are reports that recommend connecting a three-unit zirconia superstructure directly to the implant body [19]. In this study, the implant-abutment joint mechanisms were used in internal butt joints. Recently, there has been a trend towards using internal taper joints or external joints at abutment level. Therefore, it is also necessary to verify torque value measurements in different connection types.

Also, zirconia was selected as the material for the final restorations owing to recent developments in metal-free and digital dentistry. Zirconia has a higher elastic modulus than metal alloys and is a material with less distortion. Therefore, torque is more likely to occur in the screw part when strain stress occurs. However, conventional metal frameworks are elastic and can compensate for stress during strain. Therefore, the torque generation is reduced, and a significant difference may not be observed in evaluations using the accumulated torque. In particular, polymethyl methacrylate (PMMA), which is susceptible to such effects, is still used in temporary restorations. Therefore, material-specific measurement studies should be conducted.

## Conclusion

With the limitations of this study, we can conclude that measurement of the torque and evaluation of the accumulated torque during the installation of screw-retained superstructures allowed numerical and objective evaluation of the passive fit and misfit. Moreover, a significant difference was observed in the accumulated torque when the misfit was 150 and 200 μm. For misfits of 150 and 200 μm, an increase in the torque was observed in the middle phase of the measurement period.

## Data Availability

All data generated or analyzed during this study are included in this published article.
